# Tobacco Use Patterns in Five Countries During the COVID-19 Lockdown

**DOI:** 10.1093/ntr/ntaa097

**Published:** 2020-05-27

**Authors:** Derek Yach

**Affiliations:** Foundation for a Smoke-Free World, New York, NY

In their recent piece, Klemperer et al.^1^ explore how motivations to quit combustible and electronic cigarettes (e-cigs) changed in response to concerns about increased risk of infection by the novel coronavirus. We offer complimentary findings from our online survey, which was conducted during a similar time period (April 4–14, 2020).

We surveyed 6800 combustible and e-cig users under a variety of lockdown measures across five countries: Italy, India, South Africa, the United Kingdom, and the United States.^2^ In the United States, we restricted our target population to California and New York given the trajectory of the virus’ spread in these states. We aimed to find out whether the lockdown affected the physical and emotional well-being of nicotine users, whether usage behaviors changed, and whether altered usage patterns affected others. At the time of the survey, Italy, India, and South Africa had restricted outdoor exercise; but in all countries surveyed, essential outdoor activities like going to a grocery store or pharmacy were permitted. South Africa was the only country to ban the sales of alcohol, tobacco, and vaping products.

The Klemperer et al. sample mainly consists of dual users who do not typically use tobacco or e-cigs every day. In our sample, the largest group in all countries was comprised of individuals who exclusively smoke combustible cigarettes. We surveyed many dual- and poly-product users, but only a small group of exclusive vapers. This distribution likely represents an urban population, especially in South Africa and India. Several methodological reviews have shown that nonrandomized online samples may not reflect the general population.^3^

We observed that e-cig consumption has marginally increased during lockdown; however, the types of products used and the frequency of that use remain virtually unchanged. In India, a large part of the surveyed population believe that smoking and vaping increase the risk of contracting COVID-19. In Italy, more than half believe that there is no relation between COVID-19 risk and smoking or vaping. Results from the other countries were less stark, although vaping was usually considered less of a risk than smoking combustible cigarettes. In a large proportion of users, the desire to quit smoking or vaping for health reasons has been affected by direct experience of the COVID-19 outbreak. In the United Kingdom and the United States, this proportion was twice as high among participants living lived in a household where someone had tested positive for the virus. Participants from such households in all countries also expressed that the lockdown had a more negative impact on their mental health.

We found that slightly more users in our study had attempted to quit all nicotine and tobacco products than in the Klemperer et al. study. This may be because their study asked specifically about quit attempts in order to reduce harm from COVID-19, whereas we asked about general quit attempts during the lockdown. Therefore, we might have also captured attempts that were made for other reasons, such as restricted product access or increased difficulties in product consumption during lockdown.

Despite differences in lockdown regulations, many of our results are consistent across countries. Most nicotine consumers report using nicotine products as their main stress and anxiety coping mechanism. Survey respondents were concerned about becoming ill with the virus, losing their jobs, and dealing with stress. Despite constant consumption trends, many exclusive combustible cigarette smokers have been buying more cigarettes than usual, triggered by fear that stores might run out of stock or be closed during lockdown. These findings are in line with market reports of stockpiling behavior.^4^ Smoking in the home increased in Italy and India among exclusive combustible cigarette smokers, which could increase secondhand smoke exposure for nonsmoking family members. Among exclusive e-cig users, no change was observed in rates of in-home vaping during the lockdown compared with pre-COVID-19 habits.

Our study complements and confirms the Klemperer et al. study in many respects. Follow-up studies must occur after lockdown measures are lifted to evaluate the long-term consequences that lockdown measures will have on smoking and vaping behavior.

## Supplementary Material

A Contributorship Form detailing each author’s specific involvement with this content, as well as any supplementary data, are available online at https://academic.oup.com/ntr.

ntaa097_suppl_supplementary_Taxonomy_form

## Funding

This survey was funded by the Foundation for a Smoke-Free World.

## Declaration of Interests

None declared.

## References

[1] Klemperer EM, West JC, Peasley-Miklus C, Villanti AC Change in tobacco and electronic cigarette use and motivation to quit in response to COVID-19. Nicotine Tob Res. 2020;ntaa072:1–2. doi:10.1093/ntr/ntaa072 PMC7197523 32343816

[2] Foundation for a Smoke-Free World. *COVID-19 Social Distancing and Stay-at-Home Policies Lead to Increased Dependence on Tobacco as Stress Coping Tool.* https://www.smokefreeworld.org/newsroom/covid-19-social-distancing-and-stay-at-home-policies-lead-to-increased-dependence-on-tobacco-as-stress-coping-tool/. Updated May 11, 2020; Accessed May 20, 2020.

[3] Bethlehem J Selection bias in web surveys. Int Stat Rev. 2020;78(2):161–188. doi:10.1111/j.1751-5823.2010.00112.x

[4] Nilsson P, Middlehurst C, Evans J Smokers stock up on tobacco and nicotine. Financial Times. https://www.ft.com/content/362d7d51-6561-493b-a29b-72784157cca7. Published April 4, 2020; Accessed May 4, 2020.

